# Perspectives for prevention reporting by federal states

**DOI:** 10.17886/RKI-GBE-2017-092

**Published:** 2017-08-30

**Authors:** Uwe Saier

**Affiliations:** Office for Health and Consumer Protection Hamburg Public Health Department, Unit Health Data and Health Promotion

## Abstract

Prevention reporting is regulated by Book V of the SGB (German Social Code, § 20d). Reporting focuses on collecting data on target groups, access paths and expenditure, but also on the analysis and evaluation of the impact of disease prevention and health promotion measures (impact evaluation). This article focuses on possible solutions to the specific challenges faced by reporting.

## The national prevention report and its legal basis

Book V of the SGB (German Social Code, § 20d) provides the legal basis for Germany’s National Prevention Strategy. Uniform basic recommendations on health promotion and disease prevention for Germany that apply to all carriers are at the heart of the strategy, as is a mandate to report on developments. These basic recommendations instruct those institutions organised within the National Prevention Conference to agree on shared goals, priority fields of action and target groups. Every four years this involves the publication of a prevention report (with the first report due on 1 July 2019) which will be provided to the Federal Ministry of Health. The federal states will have the opportunity to contribute findings from federal state level health reporting to this report.

The federal framework recommendations, which were adopted on 19 February 2016, defined three joint targets concerning the Preventive Health Care Act: Grow up healthy, Living and working healthy and Healthy ageing. Accordingly, the national prevention report should provide data on measures in these fields regarding target groups, access paths, quality assurance, co-operations and expenditure.

## Key activities in Hamburg

Approaches and partnerships that proved successful in the context of Hamburg’s Pakt für Prävention (Framework agreement on Prevention) [1], which includes a life stage centred framework programme, as well as the Arbeitsschutz Partnerschaft (Partnership for Occupational Safety) [2], which includes disease prevention activities in and together with companies, schools and vocational schools, are to continue. An important focus is placed on documentation, evaluation and quality assurance. Further points of reference are data from federal state level health reporting, the occupational safety and health report produced by the Federal Institute for Occupational Safety and Health [3] as well as the results and targets of the Joint German Occupational Safety and Health Strategy [4]. Every four years, analogous to the national prevention report, Hamburg will produce a health promotion and prevention report.

## Implications for health reporting

As indicator selection for health reporting has always recognised the potential of disease prevention measures to influence results, the demands placed on reporting, quality assurance and use of health care reporting results laid out in the Preventive Health Care Act do not, per se, imply a paradigm shift. However, the Act does emphasise a perspective that is increasingly guided by the principle of monitoring the effectiveness of measures as a form of impact evaluation.

## Challenges and approaches to solutions

When measuring health, it is important to recognise that subjective factors are at least as important as objective measurements. Among the particular challenges prevention reporting faces is the fact that the effects of interventions are hard to identify and that the potential effects of measures to promote health can usually only be measured in the long-term. Moreover, the results from earlier evaluations are generally not standardised and therefore hardly comparable.

Evaluating the effectiveness of disease prevention and health promotion measures crucially relies on the capacity to first identify those factors that indicate the success of such measures. We must identify and document the structures and processes behind effective health promoting interventions, (plausibly) attribute them to successful interventions and, at a later stage, apply these as models of best practice to further intervention approaches. The goal would be to develop evaluation guidelines that are decided upon through stakeholder consensus and, where possible, to establish indicators for evaluation. Here specific indicators for the respective fields of action and approaches should be applied to measure the quality of processes and results in key measures and projects.
